# Stabilization of Protein Interactions through Electrospray Additives in Negative Ion Mode Native Mass Spectrometry

**DOI:** 10.1021/acs.analchem.5c00757

**Published:** 2025-05-15

**Authors:** Alexander Stevens, Mia L. Abramsson, Mark T. Agasid, Timothy M. Allison, Michael Landreh

**Affiliations:** † Department of Cell and Molecular Biology, 8097 Uppsala University, 751 24 Uppsala, Sweden; ‡ Department of Microbiology, Tumor and Cell Biology, Karolinska Institutet, 171 65 Solna, Sweden; § AgResearch Ltd., Lincoln 7608, New Zealand; ∥ Biomolecular Interaction Centre, School of Physical and Chemical Sciences, University of Canterbury, Christchurch 8140, New Zealand

## Abstract

Native mass spectrometry (nMS) is routinely used to monitor ligand binding in protein complexes. However, such interactions are easily distorted by unwanted dissociation during desolvation and ionization. This effect can be mitigated by chemical additives that modulate the ion charge, which results in lower-energy ions and better-preserved interactions. While positive-mode is the most used ionization approach, the use of negative-mode ionization has attracted attention due to its surprising ability to reduce the dissociation of some protein complexes. We therefore investigated whether charge-reducing compounds that stabilize proteins in positive ionization mode, such as imidazole, acetonitrile, and trimethylammonium-*N*-oxide, affect the charge and stability of complexes in negative ionization mode. Using the complex between myoglobin and its heme cofactor as a model system, we find that charge reduction efficiency is not maintained between positive and negative polarity, as expected for different charge reduction mechanisms. We also suggest that charge and stability correlate in positive and negative ion modes. Notably, the strongest stabilizing effects were observed for compounds that form electrospray adducts, which can dissociate during in-source activation and cool the desolvated ions. Together, the effects of charge-modulating additives suggest that relative ion stabilities may be directly comparable between polarities, although the protective effects of evaporative cooling have to be considered. We demonstrate the usefulness of the approach to detect cooperative stabilization of lysozyme by two epigallocatechin-3-gallate molecules, which requires negative ion mode and charge reduction. The strategic use of chemical additives in negative-mode ionization experiments may therefore be important for preserving complexes for analysis.

## Graphical abstract

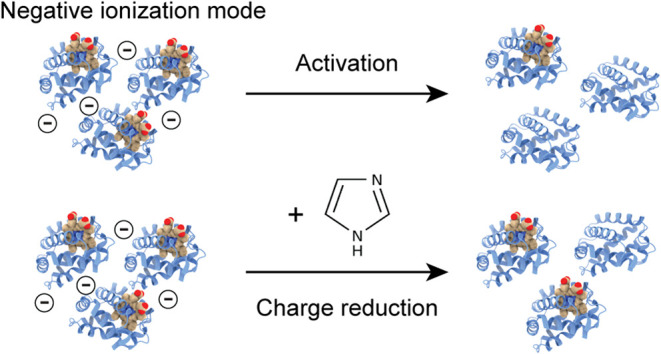

## Introduction

Native mass spectrometry (nMS) can be applied to inform about the interactions in biomolecular complexes in near-native states.^1,2^ In this method, complexes are subjected to (nano)­electrospray ionization (nESI) from solutions in the physiological pH range and gently transferred to the gas phase to preserve structural and functional integrity. nMS is regularly employed to measure protein and nucleic acid interactions, providing insights into their stoichiometry, topology, and dynamics.^3^

A critical challenge in nMS is the destabilization of large complexes upon their transfer to the gas phase. nESI produces multiply charged ions, where the number of charges is determined by the Rayleigh limit.^4^ Highly charged species are destabilized by Coulombic repulsion and high-energy collisions with residual gas molecules in the ion source of the mass spectrometer. Charge-reduction techniques, such as ion–ion reactions (e.g., proton transfer reactions) or ion-neutral collisions, play a pivotal role in mitigating this issue. Reducing the net charge of the complex enhances its stability in the gas phase because it undergoes lower acceleration and thus experiences lower-energy collisions with gas molecules. In addition, charge reduction reduces Coulombic repulsion between the ESI charges.^5^ Charge reduction has enabled the detection of intact protein–drug assemblies,^6,7^ stabilization of labile protein–lipid complexes,^8,9^ and the preservation of compact gas-phase conformations.^10,11^ For clarity, we refer to charge reduction as a reduction in the number of charges (e.g., 4+ to 3+ or 4– to 3−), not a reduction in net charge (e.g., 4+ to 3+ or 3– to 4−).

The vast majority of nMS studies employ ionization in positive mode, where protons are produced at the electrospray interface and are eventually deposited on the protein surface as charge carriers.^4,12^ An alternative approach is to use negative-mode ionization for the nMS of proteins. Protein complexes ionized in negative ion mode acquire a slightly lower number of charges than in positive mode.^13,14^ They retain native-like cross sections in ion mobility experiments,^15^ and negative ionization can preserve specific membrane protein–lipid interactions.^16^ Furthermore, a comparison of surface-induced dissociation of multiprotein complexes in positive and negative ion modes revealed that native subunit connectivities are not affected by the change in polarity.^17^ In summary, the lower number of charges in negative ion mode appears to stabilize noncovalent interactions.

Stabilization of protein complexes in positive ionization mode is usually achieved with charge-reducing chemical additives, which are either added to the electrospray solution or introduced as vapors in the ion source of the mass spectrometer.^18^ Common charge-reducing compounds used to protect weak interactions are acetonitrile,^19^ imidazole and its derivatives,^20^ triethylammonium,^21^ and poly­(ethylene glycols) (PEGs).^22^ Trimethylamine oxide forms adducts that are highly charge-reducing when removed by gas-phase activation.^9,20,23,24^ Imidazole derivatives and ethyl amines reduce the number of charges of proteins also in negative ion mode.^16,25^ Importantly, some charge-reducing reagents form clusters with the analyte ions, whose dissociation can additionally provide collisional cooling of a complex as it is accelerated into the vacuum region of the mass spectrometer.^18^

Together, the observations that (1) the lower charge acquired in negative ion mode stabilizes protein complexes^13,15–17^ and (2) complex stability and charge can be tuned by electrospray additives^6,18,8^ raise the question of whether the strategies can be combined to minimize complex dissociation in negative ion mode.

## Methods

### Sample Preparation

All chemicals were purchased from Sigma unless noted otherwise. Myoglobin and lysozyme were dissolved in dH_2_O and buffer-exchanged into 100 mM ammonium acetate, pH 6.9, using Zeba Spin columns (Thermo Fisher Scientific) with an approximate final protein concentration of 5 μM. Acetonitrile vapors were introduced into the ion source by placing a 50 mL Falcon tube cap filled with 3 mL of acetonitrile below the capillary holder. C8E4 (Anatrace) was added to a final concentration of 16 mM (2× CMC). The final concentrations of TMAO and imidazole were 50 and 40 mM, respectively, and the sulfolane concentration was 207 mM. EGCG was added to a final concentration of 40 μM.

### Native Mass Spectrometry

nESI capillaries (borosilicate, medium) were purchased from Thermo Fisher Scientific (Cat. Number ES380). Mass spectra were acquired on a Q Exactive Plus instrument (Thermo Fisher Scientific), modified for high-mass analysis (MS Vision, NL). The resolution was set to 17 500. The capillary voltage was set to +1.5 kV in positive and −1.5 kV in negative ion mode. The source temperature was 30 °C and the fore vacuum (ion source) was 2 mbar. In-source activation was performed by ramping the in-source fragmentation voltage from 0 to 200 V. Data were analyzed by using Xcalibur 2.2 (Thermo Fisher Scientific) and Microsoft Excel.

### Molecular Docking

Docking of EGCG to lysozyme was performed using the SwissDock server (www.swissdock.ch).^26^ PDB entry 1LYS (hen egg white lysozyme) was used as the receptor. Settings were: Box center: −3–21–38, box size: 30–34–36, sampling exhaustivity: medium, number of random initial conformations: 8, cavity prioritization: buried. Output files were visualized using UCSF ChimeraX V1.7.1.^27^

## Results

To answer whether charge reduction can stabilize complexes in negative mode, we turned to holo-myoglobin with its heme cofactor, a common system to assess the gas-phase stability of protein complexes, which yields well-resolved spectra in positive and negative ionization modes.^14,28^ We first compared heme dissociation caused by in-source activation in positive and negative ion modes (Figure 1). In agreement with previous studies,^29,30^ in positive mode, the heme group, which is predominantly Fe­(III), was lost as a positively charged ion in response to activation, leading to the appearance of apo-myoglobin with an average charge of 7.0+, whereas holo-myoglobin maintained an average charge of 7.9+. In negative ion mode, we detected holo-myoglobin with an average charge of 6.5–. In-source activation produced less apo-myoglobin than in positive ion mode (Figure 1).^30^ Earlier studies suggested that positively charged Fe­(III) heme binds tightly to negatively charged myoglobin ions,^30^ however, we observed no shift to a higher number of charges (e.g., from 7– to 8−) upon dissociation, suggesting that heme is dissociated as a neutral species.^29^ The higher stability in negative ion mode is potentially related to the lack of Coulombic repulsion between protein and heme, as well as the overall lower charge-reducing unfolding, in accord with expectations about the dependence of ion energy on charge magnitude.^16^ In addition, these charges may be distributed differently on the protein’s surface due to the spacing of ionizable groups,^31^ which may provide additional stabilization.^32,33^

**Figure 1:**
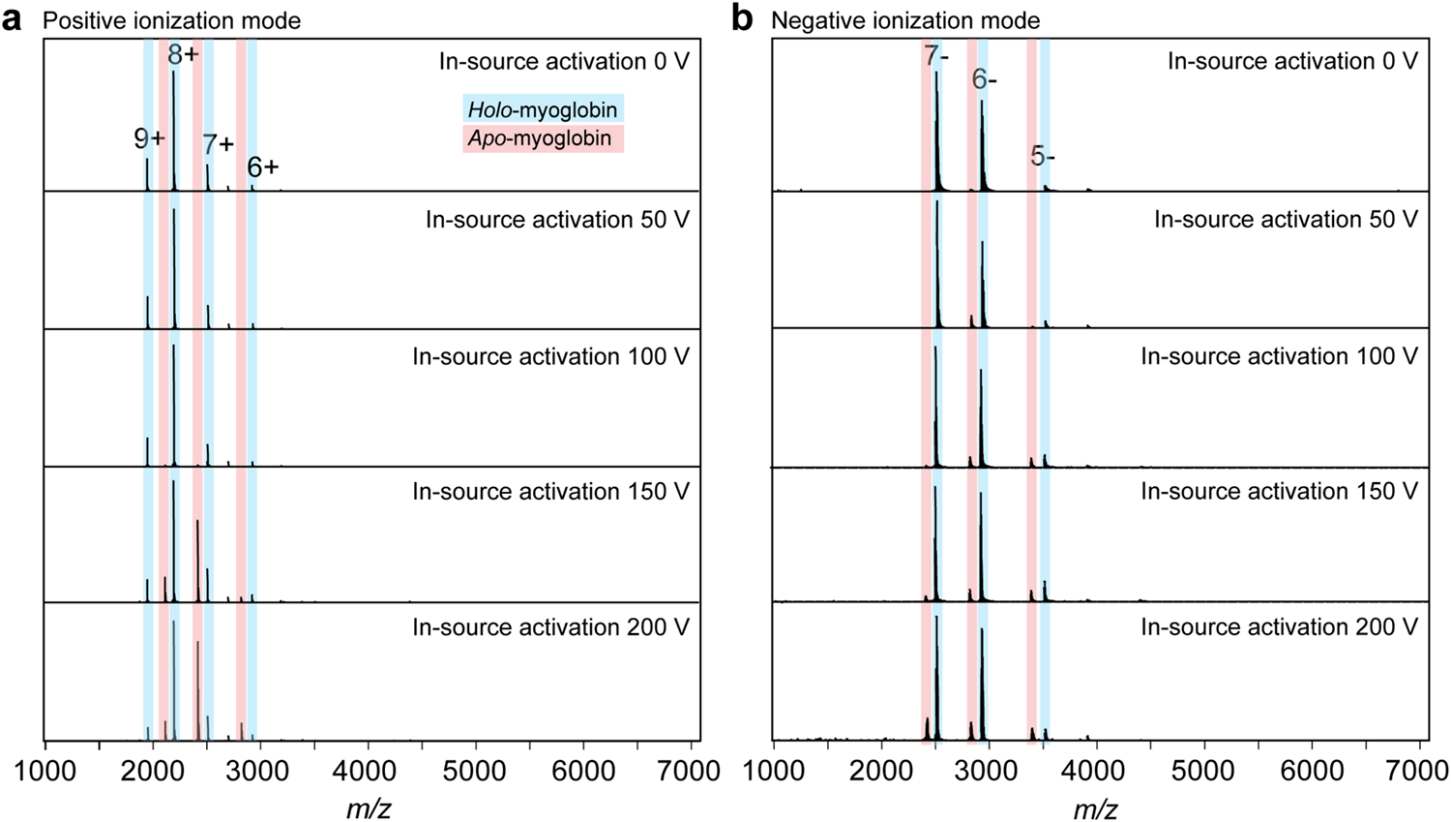
In-source dissociation of holo-myoglobin in positive and negative ionization modes. (a) Increasing the in-source activation in positive ionization mode resulted in the dissociation of the heme group as a singly charged ion and the appearance of apo-myoglobin ions with the charge lowered by one. (b) In-source activation in negative mode caused only minor heme dissociation and a progressive charge reduction for holo- but not apo-myoglobin at higher activation voltages.

Having established polarity-dependent charge state signatures and heme dissociation patterns in ammonium acetate, we next compared how myoglobin ion charge states were affected by charge-modulating additives in the positive ion mode. Specifically, we tested acetonitrile, the PEG detergent C8E4, imidazole (which has been identified as charge-reducing also in negative mode),^16,25^ and trimethylammonium-*N*-oxide (TMAO), as well as the supercharging reagent, sulfolane, which increases ion charge (Figure 2a).^34^ Acetonitrile was introduced into the source region as vapor, imidazole and TMAO were added to the electrospray solution at a concentration of 50 mM, sulfolane at 207 mM (2.5% v/v), and C8E4 at 16 mM (2× CMC). In positive mode, we observed progressive charge reduction in the order acetonitrile > C8E4 > imidazole > TMAO. We detected a pronounced increase in ion charge for sulfolane, consistent with supercharging.^35^ The detergent C8E4 forms micelles in solution, which can attach to soluble proteins and can be dissociated inside the mass spectrometer.^36^ Here, we had to raise the in-source activation to 50 V to dissociate detergent clusters and detect myoglobin. Interestingly, we observed that the degree of charge reduction increased with increasing in-source activation (Figure S1), which led us to speculate that C8E4 adducts dissociate with a charge. Such a mechanism has been established for TMAO, which reduces ion charge by forming adducts on basic side chains and dissociating with a positive charge.^20,23,24^ In the presence of TMAO, we observed a mixture of TMAO and sodium adducts bound to myoglobin (Figure S2). The number of adducts was higher for lower charge states, as expected, because of the lower activation they experience during desolvation. As expected, in-source activation resulted in additional charge reduction and adduct removal (Figure S2).

**Figure 2:**
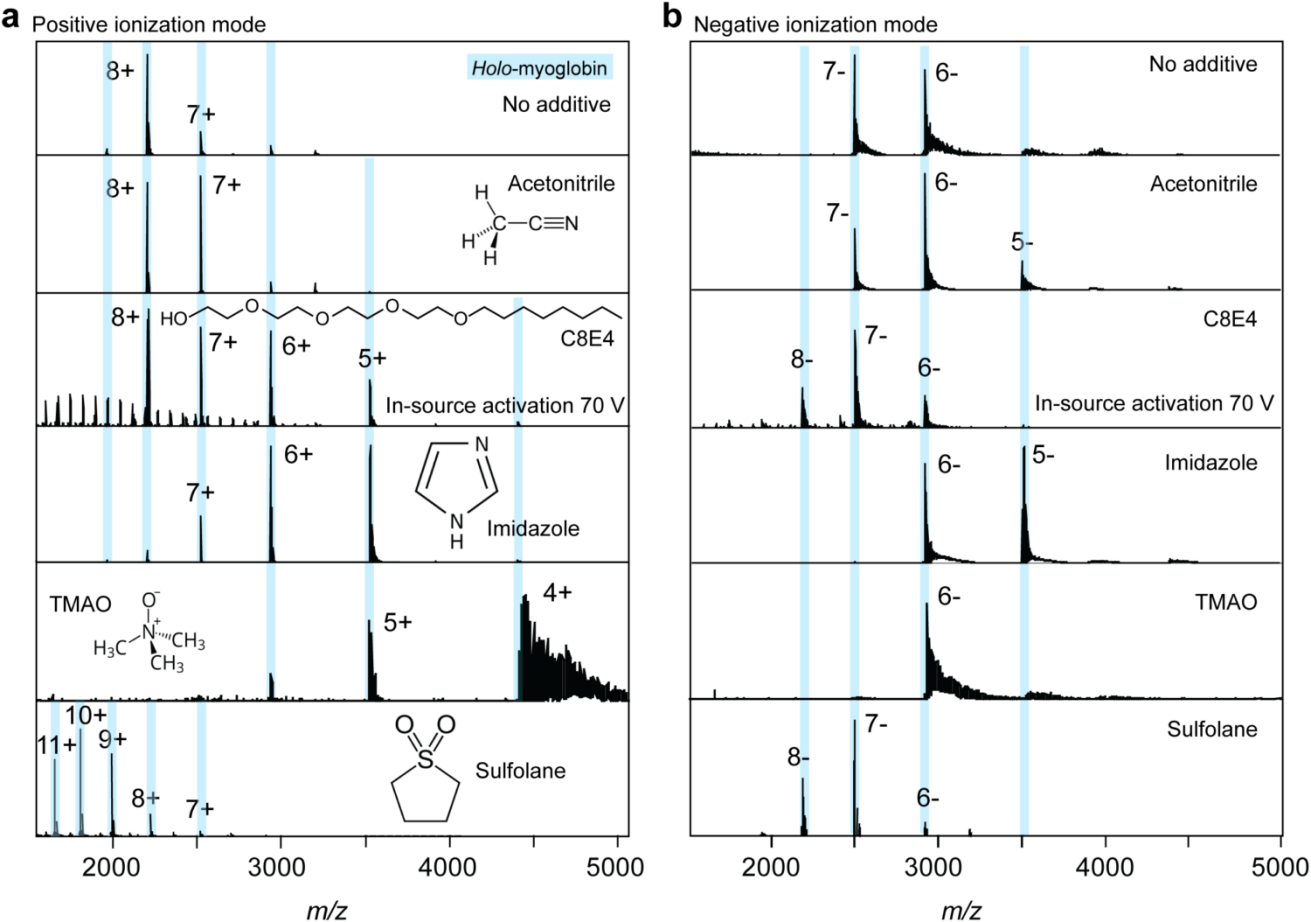
Effects of charge-modulating compounds differ between positive and negative ionization modes. (a) Mass spectra recorded in positive ionization mode, and (b) mass spectra of the same solutions recorded in negative ionization mode. Acetonitrile vapor reduces ion charges by less than one in both polarities. Imidazole reduced the charge by 2–3 in positive and 1–2 in negative ionization mode. PEG detergent C8E4 was charge-reducing in positive ionization mode but slightly increased the ion charge in negative ionization mode. TMAO was strongly charge-reducing in positive ionization mode but only moderately in negative polarity. In the presence of C8E4, 70 V in-source activation was required for the detection of myoglobin in both polarities. In the positive mode, detergent cluster peaks were also observed.

Next, we analyzed the effects of the additives in negative ion mode (Figure 2b). Without additives, the average charge state of holo-myoglobin was 6.6–. We found that acetonitrile induced a small but notable charge reduction to 6.2–. Imidazole, by contrast, caused a pronounced charge reduction from 6.6 to 5.4, while C8E4 resulted in a small increase in ion charge to 7.1–. TMAO, which is the most potent charge reducer in positive ion mode, showed only a moderate shift from 6.6 to 5.9 but a much narrower charge state distribution of essentially only the 6– ion peak. As in positive ion mode, the addition of TMAO resulted in an adduct formation (Figure S2). The adducts represent a mixture of Na^+^, Cl^–^, as well as some, but fewer, TMAO adducts than in positive ion mode. Detection of myoglobin in the presence of 16 mM C8E4 again required in-source activation to yield well-resolved spectra (Figure S2). As described before, sulfolane slightly increased the ion charge also in negative mode,^37,38^ to a similar extent as C8E4 (Figure 2b).

We next tested the impact of the electrospray additives on the stability of the holo-myoglobin complexes by subjecting them to in-source activation. We then determined the average charge of holo-myoglobin and the fraction of apo-myoglobin (Figure 3a). In positive ion mode, we found a linear correlation between ion charge and resistance to in-source fragmentation. Reducing the average charge of holo-myoglobin from 8+ to 3+ increased the fraction of intact complexes from 50 to 98%. In-source activation with C8E4 resulted in moderate charge reduction, albeit to a lesser extent than in positive ion mode (Figures S1 and[Fig fig3]). In the presence of sulfolane, we observed protein fragmentation at 200 V and near-complete dissociation of holo-myoglobin already at 100 V of in-source activation (Figure 3a). These data illustrate that the reduction of ion charge is the predominant stabilizing factor for positively charged complexes. In negative ion mode, in-source activation resulted in less dissociation, with more than 85% of the complex remaining intact under all conditions (Figure 3b). The average charges were generally less affected by the additives than in positive ion mode, ranging from −7.1 to −5.7. We observed a similar correlation between the average charge and the dissociation of holo-myoglobin as in positive ion mode. The addition of imidazole, the most charge-reducing agent in negative ion mode with an average charge of −5.7, resulted in a slight increase in the fraction of intact myoglobin from 19 to 16%. With sulfolane, we observed an increase in apo-myoglobin from 19 to 36%, consistent with the higher average charge of −7.1.

**Figure 3:**
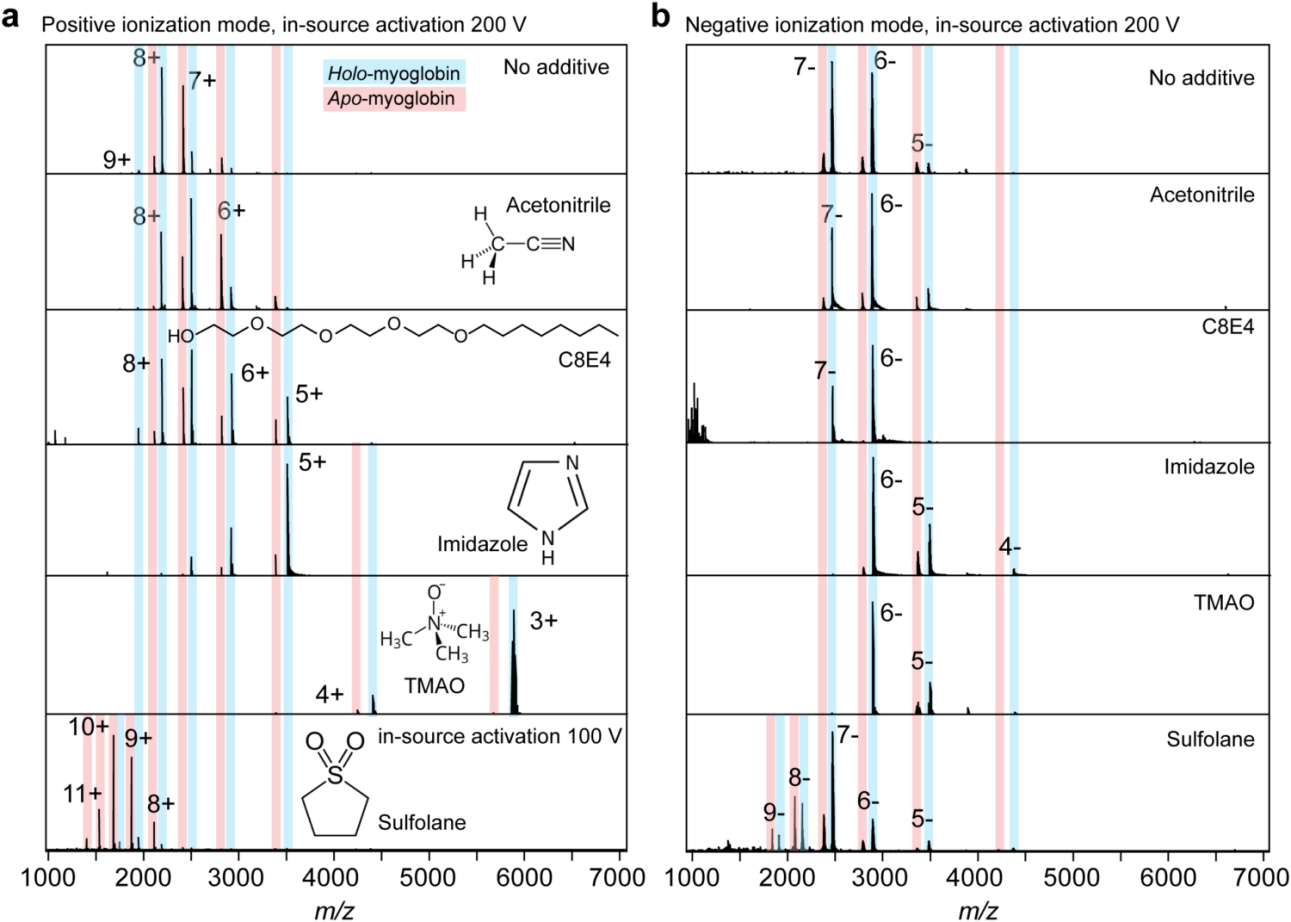
Ion charge affects the in-source dissociation of myoglobin in positive and negative ionization modes. (a) Mass spectra of myoglobin in the presence of charge-modulating agents and subjected to maximum in-source activation (200 V) in positive and (b) negative ionization mode. Acetonitrile was introduced as a vapor in the ion source region of the mass spectrometer. Imidazole, C8E4, TMAO, and sulfolane concentrations were 40, 16, 50, and 207 mM, respectively.

Interestingly, we observed virtually no dissociation in the presence of TMAO and C8E4 despite having essentially the same charges as myoglobin with imidazole and acetonitrile (Figure 3b). These observations suggest that the increased stability is, in these cases, not caused by a reduction in Coulombic repulsion. Lastly, we also tested the effect of 2× CMC lauryl dimethylamine-*N*-oxide (LDAO), which has a hydrophobic acyl chain and a TMAO headgroup and reduces ion charge in positive mode.^9,23^ As expected for a “harsh” detergent, LDAO, in general, caused near-complete denaturation of myoglobin, resulting in broad charge state distributions and only a minor holo-myoglobin population (Figure S3). More specifically, in positive ion mode, we observed pronounced charge reduction and near-complete loss of holo-myoglobin in response to in-source activation. In negative ion mode, LDAO strongly promoted the formation of mixed salt adducts, resulting in similar peak broadening as seen for TMAO. Interestingly, this fraction of holo-myoglobin was preserved in negative ion mode, even at 200 V in-source activation (Figure S3).

To test whether the protective effect of charge reduction in negative mode could provide new insights into protein–ligand interactions, we analyzed the association between lysozyme and epigallocatechin-3-gallate (EGCG). Aggregation of lysozyme into amyloid fibrils can give rise to hereditary non-neuropathic lysozyme amyloidosis. In vitro, fibril formation of hen egg white lysozyme, a functional and structural homologue of human lysozyme, can be inhibited by EGCG.^39^ Molecular docking studies identified a putative binding pocket in folded lysozyme where EGCG interacts via hydrogen bonding with arginines 61 and 112, as well as π–π interactions with tryptophan 62 and 63.^40^ We reasoned that the absence of charge interactions makes the complex insensitive to ionization polarity but not particularly stable in the gas phase. Mass spectra in positive and negative ion modes showed binding of one, and to a lesser extent, two EGCG molecules to the 7+ and 6+ and −6 and −5 ions, respectively (Figures S4 and 3a). Increasing the in-source fragmentation from 50 to 100 V completely dissociated these complexes (Figures S4 and 4a). Next, we added 50 mM imidazole to the ESI solution, resulting in a moderate charge reduction from −5.7 to −5.3 (Figure 3b). At 50 V in-source activation, we did not observe a notable change in EGCG retention compared to noncharge-reduced complexes. However, upon increasing the in-source fragmentation to 100 V, we found that the −5 and −6 ions both preferably retained two EGCG adducts, whereas the previously dominant 1:1 complexes were dissociated completely (Figure 4b). Importantly, charge reduction in positive mode did not result in increased stability of the 1:2 complex for any of the charge states (Figure S4). These data indicate that the 1:2 complex has a marginally higher stability, which can be detected through charge reduction in the negative mode. To better understand the basis for this observation, we performed docking of EGCG to lysozyme using SwissDock.^26^ In addition to the known binding pocket, we observed a second, less favorable EGCG binding site on the opposite side of the protein (Figure 4c). The existence of a secondary binding site would rationalize the observation of chemical shift changes outside of the canonical binding pocket in NMR titration experiments with EGCG.^41^ Mapping this second group of affected residues on the structure of lysozyme revealed an excellent agreement with the secondary site proposed by SwissDock (Figure 4c). We speculate that occupation of both sites could lead to cooperative stabilization of the protein, favoring the retention of two EGCG molecules and thus confirming the existence of a secondary EGCG binding site.

**Figure 4:**
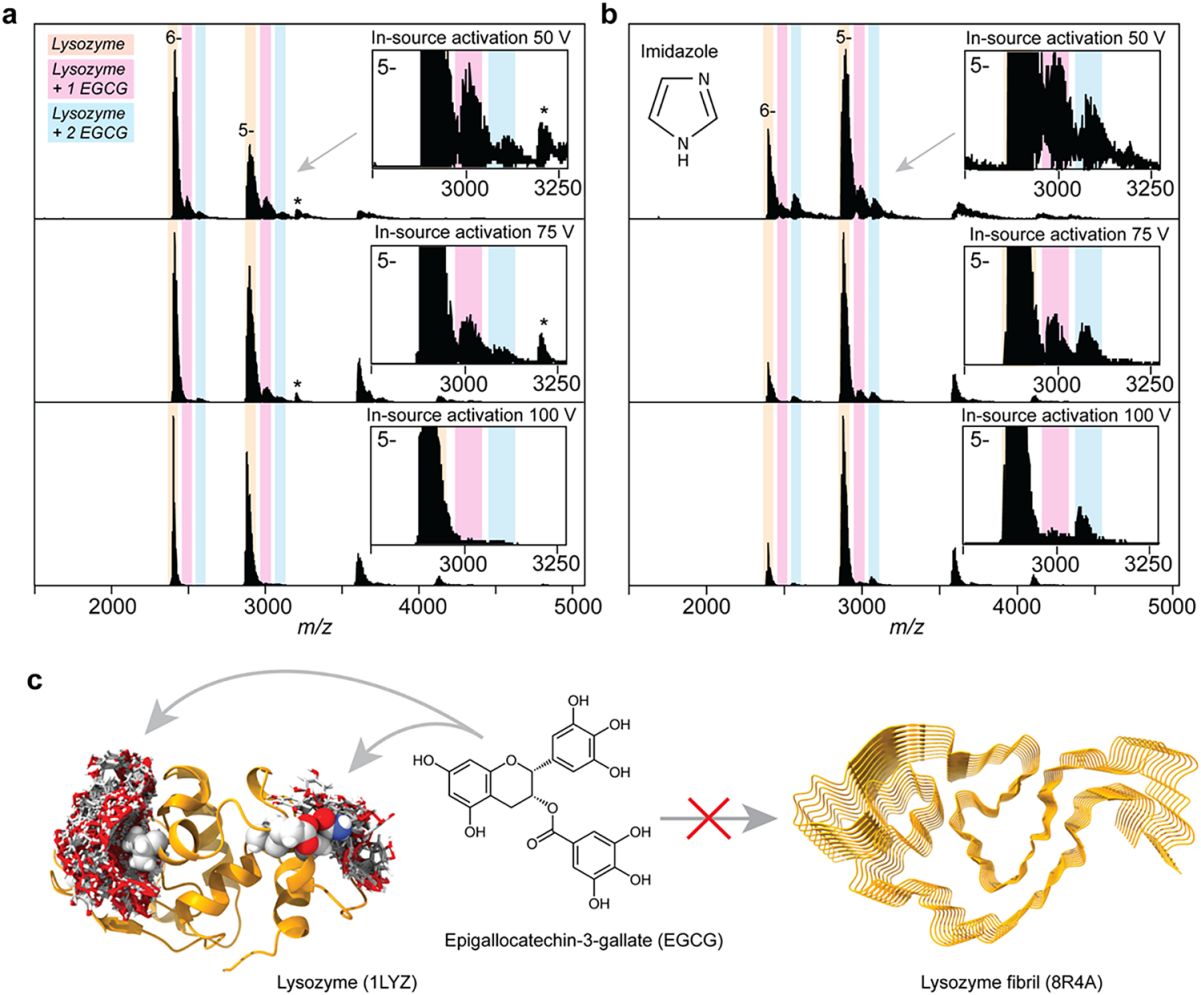
Charge reduction in negative ion mode reveals cooperative stabilization of the lysozyme-EGCG complex. (a) Negative ion mode mass spectra of lysozyme in the presence of EGCG showed retention of up to two EGCG molecules, which could be dissociated by increasing the in-source activation voltage from 50 to 100 V. Asterisks indicate a minor lysozyme dimer population. (b) The addition of 50 mM imidazole led to a moderate charge reduction. In-source fragmentation caused dissociation of the 1:1 lysozyme-EGCG complex, whereas the 1:2 complex was retained at higher activation voltages. The −5 charge state is shown as a 10× zoom in the inset. (c) The SwissDock model of hen egg white lysozyme with 30 EGCG molecules indicated the primary EGCG binding site (left) as well as a potential secondary binding site (right). Residues shown by NMR to interact with EGCG are shown as spacefill. Occupation of the primary and possibly secondary sites prevents the conversion of lysozyme into amyloid-like fibrils.

## Discussion

Although electrospray additives modulate ion charge in positive and negative ion modes, their net effect on ion charge, and likely also signal intensity,^42^ differs greatly between both polarities (Figure 2). Ogorzalek-Loo and Smith suggested that cluster formation between the protein and charge-reducing agent enables proton transfer, depending on gas-phase basicity, temperature, and ESI polarity.^43^ As a result, additives that are highly charge-reducing in positive mode may have poor charge-reduction properties in negative ion mode. TMAO illustrates this concept by forming adducts at protonated sites in positive ion mode, which dissociate with a proton to reduce ion charge^23,24^ but cannot attach to protein ions formed in negative mode (Figure S1). Imidazole, on the other hand, contains two protonation sites with basic and acidic p*K*_a_, respectively. It can therefore act as a proton acceptor in positive ion mode and a proton donor in negative ion mode, leading to charge reduction regardless of polarity.^44^ Being the most potent charge reducer in negative ion mode included here, we demonstrate its ability to preserve labile interactions using lysozyme-EGCG complexes. Its moderate charge reduction potential is sufficient to detect stability differences between 1:1 and 1:2 complexes.

Plotting the survival yield as a function of ion charge reveals a similar trend in positive and negative ionization modes (Figure 5). The connection between Coulombic destabilization and ion charge is well-established for positively charged protein complexes.^6,11^ In negative ion mode, charge-carrier emissions happen at a lower field strength, which results in a reduced number of charges on ions compared to positive ion mode.^15,31^ Imidazole, which produces a similar average number of charges in positive and negative polarity (+5.4 and −5.6, respectively), yields near-identical percentages of intact holo-myoglobin (86 and 84%, respectively) (Figure 3). The same holds true for the comparison of acetonitrile in positive and sulfolane in negative mode, which produce similar average charges (+7.0 and −7.1, respectively) and similar fractions of intact myoglobin (35 and 36%, respectively). Hong and Bush reported that the 14+ and 14– charge states of avidin tetramers display a similar, but not identical, sensitivity to collision-induced dissociation.^33^ Using chemical charge modulation, our data show that positive and negative protein ions display a similar correlation between stability and charge state. This finding implies that relative stabilities measured in negative and positive ionization modes are roughly comparable, which is of interest for binding studies with ligands that are sensitive to polarity, such as lipids.^45^ Subtle differences in stability between the same charge states in positive and negative ion modes exist (Figure 1), which may be attributed to the differences in where the charges are located.^32,33^

**Figure 5:**
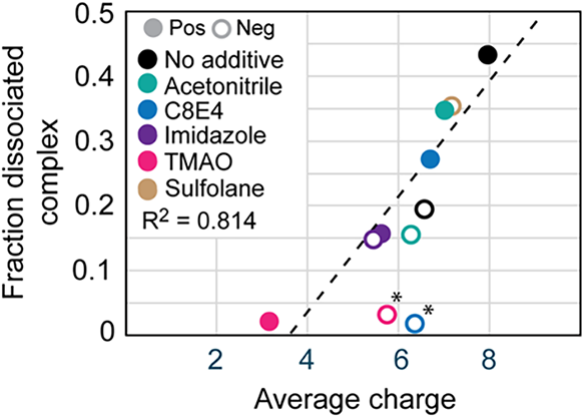
Fraction of dissociated complexes at 200 V in-source activation as a function of the average charge of holo-myoglobin shows that the degree of charge reduction generally correlates with reduced complex dissociation in both polarities. In positive polarity, the most pronounced protective effect is observed for the most potent charge reduction agents imidazole and TMAO. In negative mode, imidazole is the most potent charge reducer. No data could be acquired for sulfolane in positive mode at 200 V of in-source activation. The most protective effects were observed for C8E4 and TMAO (marked with asterisks), which was attributed to evaporative cooling rather than charge reduction. Data were fitted using a linear regression. Negative-mode data points for C8E4 and TMAO were not included in the fitting of the data (see the Discussion section).

Interestingly, we observed an increased resistance to in-source dissociation in the presence of C8E4, TMAO, and LDAO. TMAO is a strong osmolyte that stabilizes the hydration shell around a protein,^46^ which may trap cations and anions, leading to the increased salt adduct formation seen in the negative ion spectra (Figure S2). All three additives displayed activation-dependent charge reduction in positive ion mode, which likely proceeds via the dissociation of adducts as charged species. In negative ion mode, adduct dissociation was largely uncoupled from a reduction in ion charge (Figures 2b, S1, and S2). Instead, their stabilizing effects in negative mode can be attributed to the dissociation of nonspecific salt or detergent adducts, which reduces the temperature of desolvated ions through evaporative cooling that was first described for imidazole.^18,47,48^ We speculate that these factors also contribute to the preservation of the 1:2 lysozyme-EGCG complexes since the −6 ion retains two EGCG molecules in the presence, but not in the absence, of imidazole despite not undergoing charge reduction (Figure 4).

## Conclusions

Comparatively little is known about the behavior of negatively charged protein ions, although their charge and stability are governed by the same principles as for positive ions. Here, we have investigated the effect of common ESI additives that modulate the charge and stability of protein complexes in both positive and negative ion modes. Our data suggest that their effects are less pronounced in negative than in positive ion mode due to the already low charge of the native-like protein ions and the strong propensity for charge-stripping in response to activation. We can, however, delineate a similarly linear relationship between protein complex charge and stability in positive and negative ESI polarity. However, further studies are needed to establish whether the stabilities can be directly compared between both modes, mostly because of the effect of evaporative cooling.

## Supplementary Material



## Notes

Activation-dependent charge reduction with C8E4 (Figure S1); adduct formation and activation-dependent charge reduction of TMAO (Figure S2); LDAO induces myoglobin unfolding and improves heme retention (Figure S3); and charge reduction of lysozyme-EGCG complexes in positive mode does not result in differential retention of EGCG adducts (Figure S4) (PDF)

## Abbreviations

nMS: native mass spectrometry
nESI: nanoelectrospray ionization
TMAO: trimethylamine-*N*-oxide
PEG: poly­(ethylene glycol)
EGCG: epigallocatechin-3-gallate
