# Supplementary material for: Stabilization of Protein Interactions through Electrospray Additives in Negative Ion Mode Native Mass Spectrometry
Source: Anal Chem. 2025 May 15;97(20):10738–44. doi: 10.1021/acs.analchem.5c00757 (PMC12120819; doi:10.1021/acs.analchem.5c00757)
Supplement: Supplementary file 1 [file ac5c00757_si_001.pdf]

Supporting information

Stabilization of protein interactions through electrospray additives in negative ion mode native mass spectrometry

Alexander Stevens,[1] Mia L. Abramsson,[2] Mark T. Agasid,[3] Timothy M. Allison,[4] and Michael Landreh [1,2,*]

[1] Department of Cell and Molecular Biology, Uppsala University, 751 24 Uppsala, Sweden

[2] Department of Microbiology, Tumor and Cell Biology, Karolinska Institutet, 171 65 Solna, Sweden

[3] AgResearch Ltd., Lincoln 7608, New Zealand

[4] Biomolecular Interaction Centre, School of Physical and Chemical Sciences, University of Canterbury, Christchurch 8140, New Zealand

* Correspondence to Michael.Landreh@icm.uu.se

Figures S1-S4

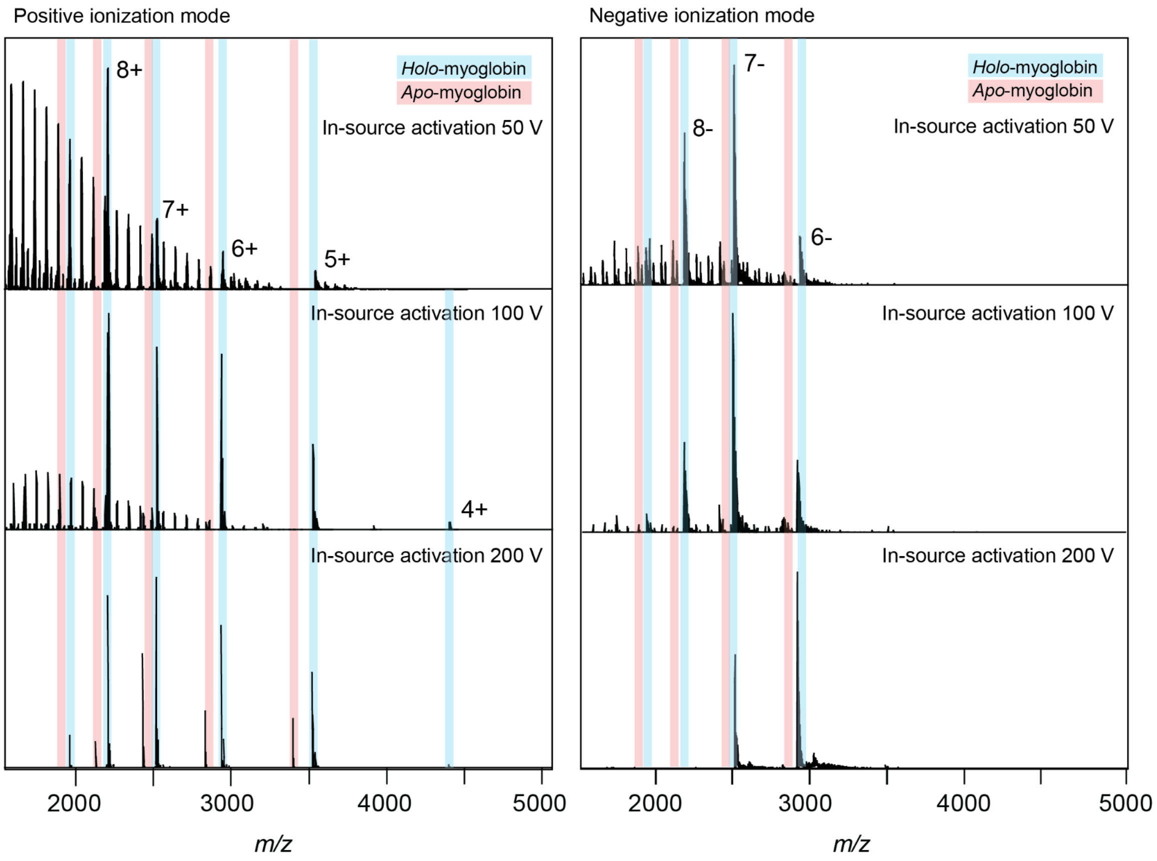

Figure S1. Activation-dependent charge reduction with C8E4. *Mass spectra of myoglobin in the presence of 2x CMC C8E4 required 50 V in-source activation to yield well-resolved spectra. Increasing the voltage to 200 V caused charge reduction in positive polarity, and to a lesser extent in negative polarity.*

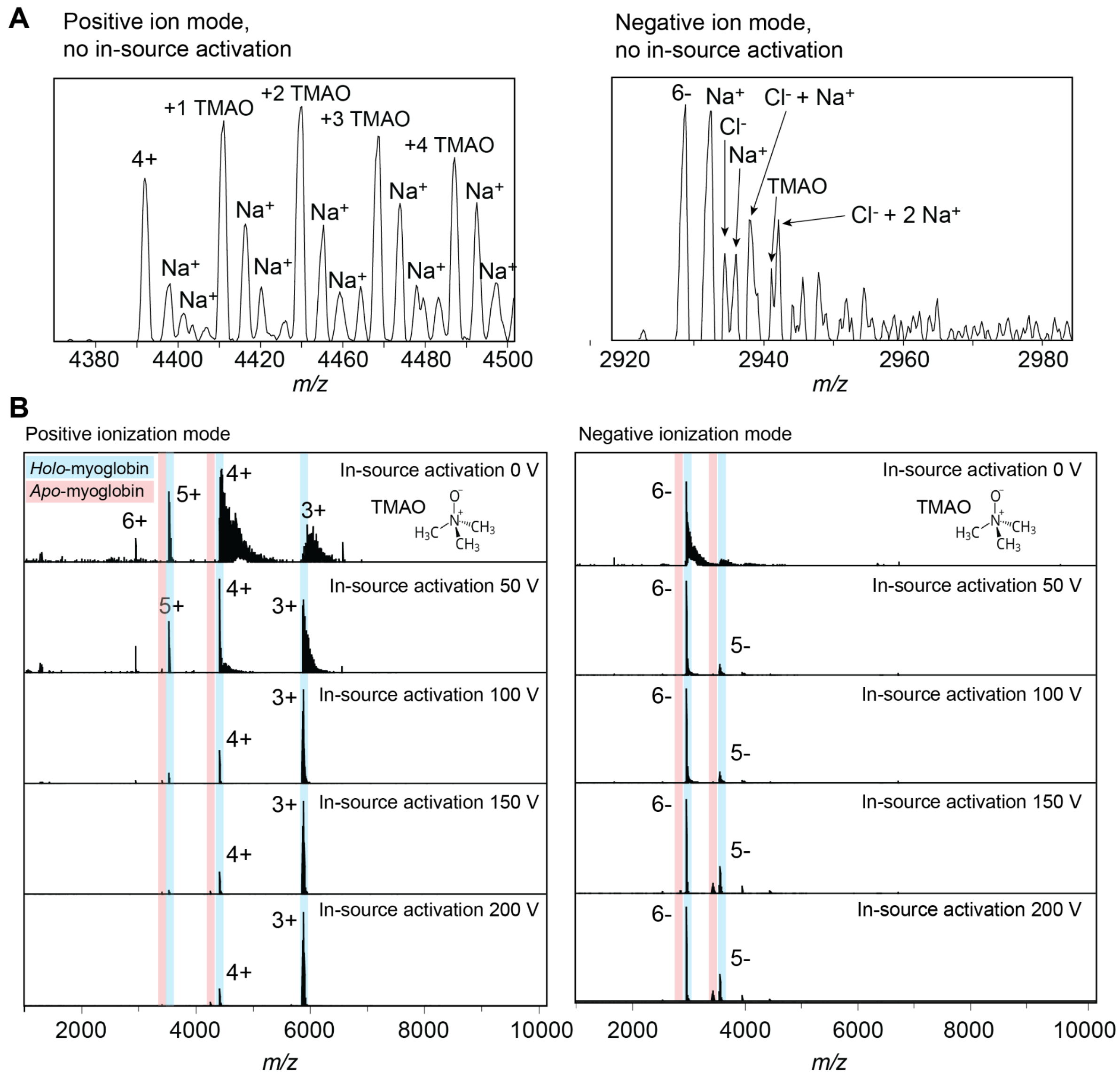

Figure S2. Adduct formation and activation-dependent charge reduction of TMAO. *(A) TMAO promotes adduct formation in positive and negative ionization mode. In positive ion mode, the adducts were primarily TMAO molecules (left). In negative ion mode, the adducts were sodium and chloride with a minor amount of TMAO adduction. Shown are the main holo-myoglobin charge states without in-source activation. (B) Raising the in-source activation resulted in adduct removal in positive and negative ion mode but caused only minor activation-dependent charge reduction in negative polarity.*

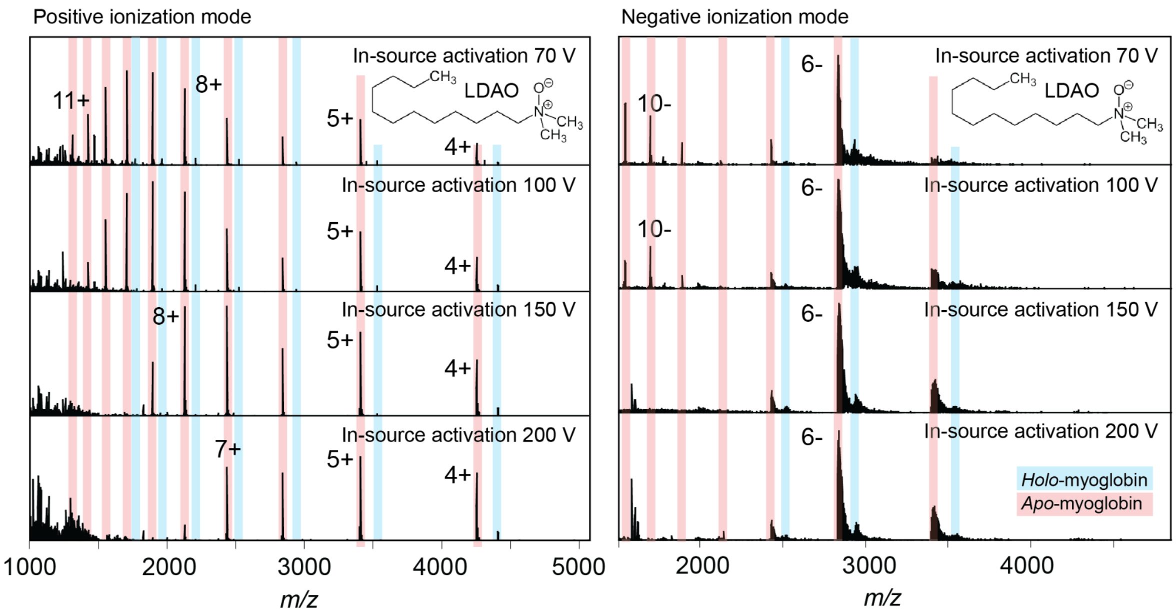

Figure S3. LDAO induces myoglobin unfolding and improves heme retention in negative ion mode. *In both polarities, LDAO induced broadening of the charge state distribution and near-complete loss of holo-myoglobin. In positive ion mode, the remaining heme complexes were dissociated by in-source activation. In negative ion mode, we found strong salt adduct formation and no dissociation of the holo-myoglobin at high activation energies.*

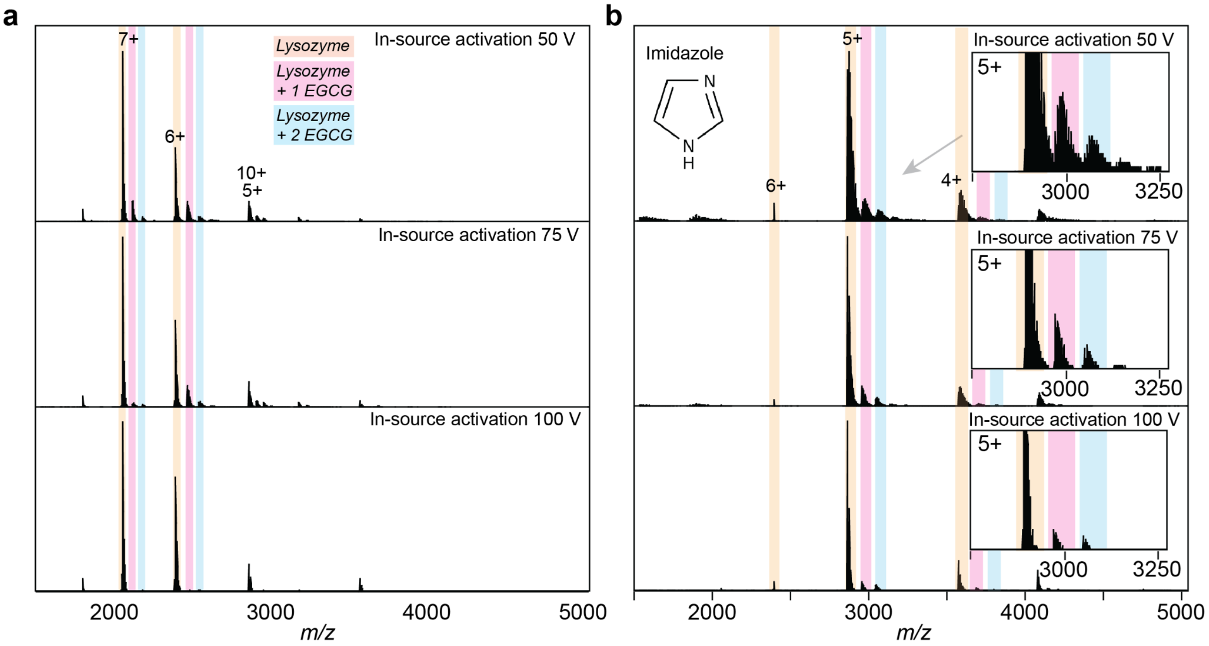

Figure S4. Charge reduction of lysozyme-EGCG complexes in positive mode does not result in differential retention of EGCG adducts. *Increasing the in-source activation in the absence* ***(a)*** *or the presence* ***(b)*** *of imidazole did not result in prominent changes of the EGCG adduct pattern. The 5+ charge state is shown as a 10x zoom in the charge-reduced spectra.*
